# eHealth Literacy 3.0: Updating the Norman and Skinner 2006 Model

**DOI:** 10.2196/70112

**Published:** 2025-03-11

**Authors:** Ariesta Milanti, Cameron Norman, Dorothy Ngo Sheung Chan, Winnie Kwok Wei So, Harvey Skinner

**Affiliations:** 1 Strategic Delivery Unit Ministry of Health - Republic of Indonesia Jakarta Indonesia; 2 Dahdaleh Institute for Global Health Research York University Toronto, ON Canada; 3 The Nethersole School of Nursing The Chinese University of Hong Kong Hong Kong China (Hong Kong)

**Keywords:** eHealth literacy, eHealth literacy definition, eHealth literacy model, eHealth literacy measurement

## Abstract

This paper advances the “eHealth Literacy 3.0” model following Norman and Skinner’s 2006 original eHealth literacy 1.0 model and Norman’s 2011 2.0 update, and introduces a corresponding revision to the eHealth Literacy Scale (eHEALS) measurement instrument (eHEALS-R).

## Introduction

Digital technology is transforming, not just evolving. A landmark definition and model of eHealth literacy was developed by Norman and Skinner [1] during the early 2000s and updated by Norman [2] when digital technology involved far fewer ways to connect and interact. Two decades later, the proliferation of digital technology has become ubiquitous. Although there are now other definitions in the literature [3-6], Norman and Skinner’s [1] version continues to be the most cited model [7,8]. An updated definition that better fits the current and foreseeable future of digital technology is overdue. This paper serves as a prompt for introducing and stimulating collaborative research on our “eHealth Literacy 3.0” model and revised eHealth Literacy Scale (eHEALS-R).

## Methods

We reviewed the published literature on eHealth literacy to consolidate the critiques and suggestions regarding Norman and Skinner’s [1] eHealth literacy definition or model. The consolidated themes were synthesized to bolster the original eHealth literacy conceptual model into version 3.0, which reflects current and anticipated use contexts.

## Results

Norman and Skinner [1] defined eHealth literacy as “the ability to seek, find, understand, and appraise health information from electronic sources and apply the knowledge gained to addressing or solving a health problem.” We have revamped the definition of eHealth literacy as “the ability to engage with digital technologies in effective, safe, and helpful ways to achieve health goals.”

**Figure 1:**
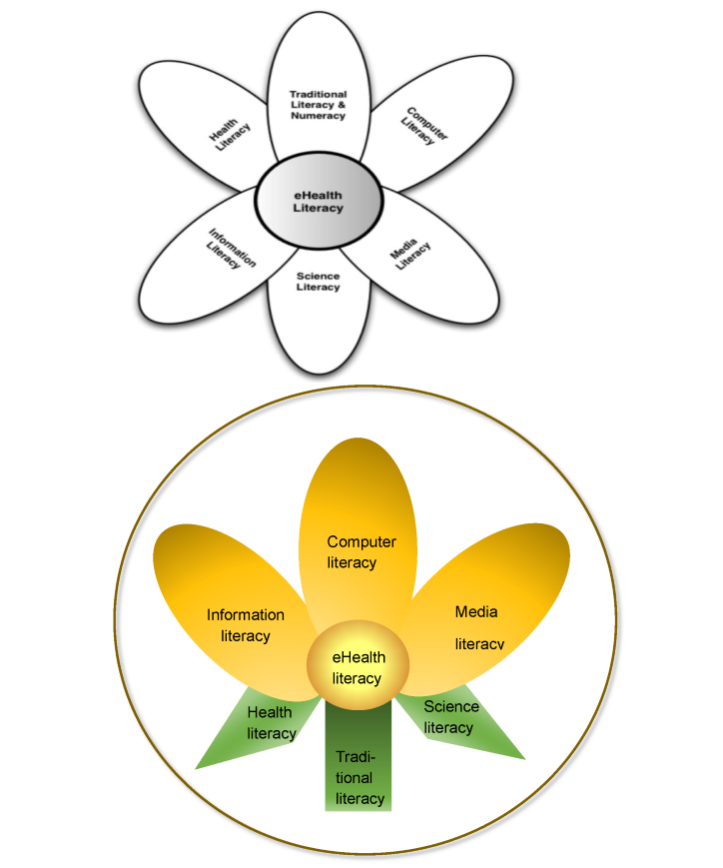
The 2006 lily model and the new lily model.

This version encapsulates an expanded concept of the original model reflecting effectiveness, safety, and helpfulness. The core idea of the original lily model is that eHealth literacy is the fused “pistil” fed by the 6 literacies (“petals”), which ties them together [1]. The new model draws more elements from the lily flower’s anatomy, whose positions and functions help to better visualize the constructs of literacies and their interrelationships (Figure 1).

Traditional literacy (referring to reading, writing, and speaking skills) embodies the stalk, from which the other 6 literacies are stemmed. Traditional literacy is a precondition, without which no other literacies can grow.

Two sepals are representative of health and science literacies. As in flowers, the sepals’ main functions are to protect and support the flowers. While health literacy implies understanding and engaging with the health system, science literacy implies understanding the key characteristics of science and how it is done. These two literacies serve as a strong ground for the other literacies above them, including eHealth literacy.

Three petals illustrate (1) computer literacy, (2) information literacy, and (3) media literacy. These petals are more pronounced in the new model, with their enhanced definitions encompassing technological advancement and contemporary paradigms. As with flower petals, which are typically the most attractive part of a flower, computer literacy is needed to keep up with the mainstream development of digital technology. Information and media literacies serve as counterparts to optimize the outcomes of the technology while mitigating the potential risks and harms posed by technological advancement.

At the center of the flower lies the pistil, representing eHealth literacy. Being held, supported, and fed by the other literacies, a person uses eHealth literacy to interact with digital technology to bear the “fruit,” that is, their health goals and needs.

Overall, the new model is shaped in a circle representing contextual factors that influence the way a person engages with digital technologies in an effective and safe manner.

Table 1 compares the original and new lily models. Modifications reflect current health technology trends with eHealth encompassing well-being, care, monitoring, and assessment, while introducing new security and privacy concerns. The modified constructs and their interrelationships are presented as initial steps, with theoretical elaboration and collaborative empirical research to follow.

**Table 1:** Comparison of two lily models. Key changes in the new model are highlighted in italics.

Element	Description	
	The 2006 lily model	The new lily model
Traditional literacy	Reading, writing, and speaking skills.	Reading, writing, and speaking skills.
Health literacy	The ability to understand and engage with the health system to make appropriate health decisions and act upon them.	The ability to understand and engage with *health source material* and the health system, make appropriate and *safe* health decisions, and act upon them.
Science literacy	The ability to understand the nature, objectives, methods, practice, limitations, and opportunities of science and/or knowledge.	The ability to understand the nature, objectives, methods, practice, limitations, perspectives and opportunities of science and/or knowledge.
Information literacy	The ability to seek, find, understand, appraise, and use information.	The ability to seek, find, understand, appraise, filter, use, and *create information ethically and safely.*
Computer literacy	The skills to use computers and to adapt to new technologies in solving problems.	*The skills to learn, adapt to, and engage with digital technologies to actively promote and support health, care and well-being.*
Media literacy	Critical thinking skills to analyze, evaluate, and use media content to form a judgment and guide actions.	*Critical thinking and conscious thinking skills* to analyze, evaluate, and use media content to form a judgment and guide actions. Critical thinking involves the use of logic to evaluate media content and guide decisions, while conscious thinking relies on self-awareness, sensory insight, clarity, and emotional control [9].
eHealth literacy	The ability to seek, find, understand, and appraise health information from electronic sources and apply the knowledge gained to addressing or solving a health problem.	*The ability to engage with digital technologies in effective, safe, and helpful ways to achieve health goals.*

## Discussion

As digital technology evolves, so does our understanding of the skills needed to navigate it for wellness and health care. While eHealth literacy’s fundamental skills remain, model 3.0 reflects our expanded digital technology ecosystem and tool set. Greater emphasis is placed on the outcome: achieving health goals effectively and safely. Research is now needed to compare and validate model 3.0. Finally, model 3.0 has been used to develop a revised 10-item version of the eHEALS [10], with items measuring each element of the model.

## Abbreviations

eHEALS: eHealth Literacy Scale
eHEALS-R: revised eHealth Literacy Scale

## Data Availability

We encourage collaboration on the evaluation and language adaptations of the new eHEALS-R (revised eHealth Literacy Scale) in diverse settings. Please contact CN and/or HS for details.
